# *Bios2mds*: an R package for comparing orthologous protein families by metric multidimensional scaling

**DOI:** 10.1186/1471-2105-13-133

**Published:** 2012-06-15

**Authors:** Julien Pelé, Jean-Michel Bécu, Hervé Abdi, Marie Chabbert

**Affiliations:** 1 CNRS UMR 6214 – INSERM 1083, Faculté de Médecine, 3 rue Haute de Reculée, Angers, 49045, France; 2 The University of Texas at Dallas, School of Behavioral and Brain Sciences, 800 West Campbell Road, Richardson, TX, 75080-3021, USA

**Keywords:** Metric multidimensional scaling (MDS), Principal coordinate analysis, R program, Supplementary elements, Evolution, Protein family, Phylogeny

## Abstract

**Background:**

The distance matrix computed from multiple alignments of homologous sequences is widely used by distance-based phylogenetic methods to provide information on the evolution of protein families. This matrix can also be visualized in a low dimensional space by metric multidimensional scaling (MDS). Applied to protein families, MDS provides information complementary to the information derived from tree-based methods. Moreover, MDS gives a unique opportunity to compare orthologous sequence sets because it can add supplementary elements to a reference space.

**Results:**

The R package *bios2mds* (from BIOlogical Sequences to MultiDimensional Scaling) has been designed to analyze multiple sequence alignments by MDS. *Bios2mds* starts with a sequence alignment, builds a matrix of distances between the aligned sequences, and represents this matrix by MDS to visualize a sequence space. This package also offers the possibility of performing *K*-means clustering in the MDS derived sequence space. Most importantly, *bios2mds* includes a function that projects supplementary elements (a.k.a. “out of sample” elements) onto the space defined by reference or “active” elements. Orthologous sequence sets can thus be compared in a straightforward way. The data analysis and visualization tools have been specifically designed for an easy monitoring of the evolutionary drift of protein sub-families.

**Conclusions:**

The *bios2mds* package provides the tools for a complete integrated pipeline aimed at the MDS analysis of multiple sets of orthologous sequences in the R statistical environment. In addition, as the analysis can be carried out from user provided matrices, the projection function can be widely used on any kind of data.

## Background

The multiple alignment of homologous sequences provides important information on the evolution and the sequence-function relationships of protein families. Two types of methods, tree-based or space-based methods, can be used to compare sequences (reviewed in [1]). Both methods depend on a multiple alignment of homologous sequences. Tree methods assume a hierarchical, binary structure of the data to infer phylogenetic relationships. On the other hand, space methods are based on multivariate analysis of a distance matrix between the sequences and do not assume a specific structure for the data. Such a method is metric multidimensional (MDS) which is a powerful method to visualize distances between elements [2-5]. MDS, also named principal coordinate analysis, starts from a matrix of distances between elements and visualizes these elements in a low dimensional space in which the distances best approximate the original distances. Applied to biological sequences, this method usefully complements phylogeny [6-11].

The completion of the genome sequencing of a wide variety of organisms has paved the way to the comparison of protein families from different species. A very interesting property of MDS is the possibility to project supplementary elements onto a reference or “active” space. The positions of the supplementary elements (a.k.a. “out of sample” elements) are obtained from their distance to the active elements [2,12,13]. This property provides a very useful tool to compare orthologous sequences to a reference sequence set. In particular, when several orthologous protein families are compared, this method can be used to visualize evolutionary drifts [9].

MDS is based on the eigen-decomposition (i.e., principal component analysis) of a cross-product matrix derived from the distance matrix [2-5] and can be performed with the default tools included in the R statistical language (e.g., *cmds* function). In addition, several R packages such as *ade4made4, adegenet*, and *vegan*[14-17] have been developed to provide multivariate analysis in the field of bioinformatics, including MDS. For example, the *dudi.pca* function in *ade4*[14] or the *wcmdscale* function in *vegan*[17] performs MDS analysis. However, the projection technique has not been widely used yet and, to the best of our knowledge, is not included in the available R packages.

Thus, we have developed the R package *bios2mds* (from BIOlogical Sequences to MultiDimensional Scaling) to provide all the tools necessary to perform the MDS analysis of multiple sequence alignments. This package includes a function that projects supplementary sequences onto a reference space and, thus, makes it possible to compare orthologous sequence sets.

## Implementation

### Main features

The *bios2mds* package has been developed in the R statistical environment. R was chosen because it is open-source, accessible under the GNU General Public License and widely used within the bioinformatics community. R packages can take advantage of functions already developed in available packages. Here, the aim of the package is to provide the tools necessary to compare orthologous sequence sets by MDS analysis, namely to analyze the active set by metric MDS to define an active space and project supplementary orthologous sequences onto this active space.

While it is possible to use available packages such as *ape*[18] and *seqinr*[19] to read sequences and compute distance matrices, we preferred to avoid too many dependencies for clarity purpose. Thus the corresponding functions were rewritten and included into the *bios2mds* package. Concerning the MDS computation, the *cmds* function provided by R gives the final results (coordinates and eigenvalues) but does not give access to the intermediary matrices that are required for the projection technique. It was thus necessary to write an MDS function from its basic equations in order to perform the projection of supplementary elements.

### Functionalities

Here, we present the main functionalities of *bios2mds*. The package provides a complete R environment for MDS analysis in the context of protein sequences. It includes functions for data import, MDS computation, clustering and visualization of the results.

#### Data import

Multidimensional scaling relies on distance matrices. The user can provide these distance matrices or compute them from multiple sequence alignments in the FASTA or MSF formats. Sequence alignments are read in with the *import.fasta* or *import.msf* functions. Several measures of distances can be computed for multiple alignments of protein sequences: the Euclidean distances based on the square roots of the difference scores [20], the distances based on the difference scores and the distances based on dissimilarity scores computed from amino acid substitution matrices. The substitution matrices provided with *bios2mds* are the JTT [21] and the Gonnet [22] matrices, the BLOSUM [23] and the PAM [24] series, along with the PHAT [25] and JTT_TM [26] matrices, more specifically developed for membrane proteins. The distances based on the difference scores can also be used for nucleotide sequences. More sophisticated measures of DNA distances can be found in several packages, such as *ade4, apeand phangorn*[14,18,27].

#### MDS computation

Briefly, given a matrix of (squared) distances between elements, MDS transforms this matrix of squared distances into a cross-product matrix whose eigen-decomposition provides the factor score matrix giving the coordinates of the elements on the principal components [2-5]. The projection of supplementary elements onto the active space depends on the matrix of distances between the supplementary and the active elements and on the factor score matrix of the active elements [2,12,13]. To make the projection possible and to facilitate subsequent data analysis especially in the case of orthologous sequence sets, we provide the *mmds* function (corresponding to the *cmds* command from R, but with a customized output) and the *mmds.project* function that performs projection of supplementary elements onto an active space.

#### Clustering

The MDS representation of the sequence space can be analyzed by *K*-means in order to find clusters. This part of *bios2mds* depends on the *amap*[28]*, e1071*[29], and *cluster*[30] packages. The *kmeans.run* function that we have developed assesses the robustness of the clustering. It depends on the *Kmeans* and the *matchClasses* functions from the *amap* and *e1071* packages, respectively. The *kmeans.run* function performs multiple runs of *Kmeans* from the *amap* package and analyzes the resulting clusters with the *matchClasses* function from the *e1071* package. The output of *kmeans.run* provides the most frequent clustering solution, with the list of the members of each cluster and their relative membership to this cluster in the different runs. The *sil.score* function performs multiple runs of the *silhouette* function from the *cluster* package. The *silhouette* function helps determining the optimal number of clusters [31]. Multiple runs with *sil.score* allow taking into account the clustering uncertainty. The *write.fasta* function allows users to retrieve the multiple sequence alignment of each cluster, in FASTA format, for further analysis.

#### Visualization

The package *bios2mds* contains graphical tools to visualize the results of the MDS analysis in R. The *mmds.2D.plot* and *mmds.3D.plot* functions are used for 2D and 3D representations, respectively, of active and supplementary elements, on the same graph, on user selected components. The *mmds.3D.plot* function is based on *plot3D* from the *rgl* package [32] that provides an interactive tool for 3D visualization within the R environment. The *col.group* function allows the use of a color code for plotting the 2D and 3D graphs, based on groupings and colors defined by the user in CSV files, for both active and supplementary elements. The *mmds.2D.multi* function visualizes the barycenters of the groups defined by the user in the 2D representation of the active elements. Finally, the *write.mmds.pdb* function allows users to export the coordinates of each element on the first three components obtained by MDS in a PDB format for visualization with a molecular graphics program, such as Pymol (http://www.pymol.org) or Rasmol (http://www.openrasmol.org). When *col.group* is used, each user-defined group corresponds to a different PDB chain number to facilitate selection and coloring.

## Results and discussion

In this section, we show and discuss the results obtained by typical MDS analyses. The input consists of non-redundant sets of non-olfactory class A G-protein-coupled receptors (GPCRs) from different species [9,33]. Two types of analysis make sense: the analysis of paralogous sequences, yielding a sequence space, and the comparison of orthologous sequences, using the projection technique.

### Analysis of paralogous sequences

**Figure 1:**
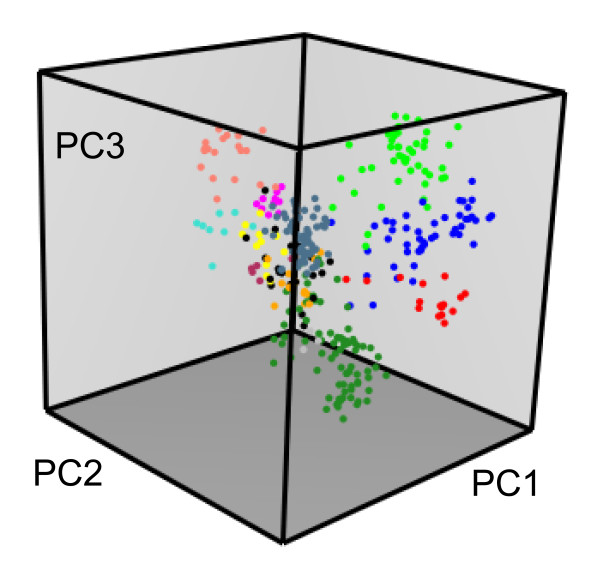
**3D representation of the GPCR sequence space.** A typical multiple sequence alignment of 283 GPCRs from *H. sapiens* was analyzed by MDS, with distances based on difference scores. The 3D space is defined by the first three components of the MDS analysis. The color code refers to the different sub-families of human GPCRs, with unclassified receptors colored in black. Plot obtained with the *mmds.3D.plot* function after coloring by GPCR sub-families with the *col.group* function.

The human set includes 283 aligned sequences of GPCRs [9]. The MDS analysis of this set provides a typical sequence space (Figure 1). In this example, the distances between sequences are equal to their difference scores and the 3D sequence space of human GPCRs is displayed with the *plot3D* command from the *rgl* package [32] that allows interactive 3D representation within the R environment. The elements are colored using the *color.group* function based on the prior knowledge of the twelve GPCR sub-families present in humans [9,33,34]. Clustering allows the grouping of these sub-families into four groups that correspond to major pathways of GPCR evolution [9].

Different distance matrices can be computed from a multiple sequence alignment. These matrices are based either on a difference score or on a dissimilarity score obtained with an amino acid substitution matrix. In MDS, the distance matrix should be Euclidean or close to a Euclidean matrix. Distances equal to the square roots of the difference scores are Euclidean [20], and the MDS analysis of the corresponding matrix gives only positive eigenvalues. Distances equal to the difference scores give negative eigenvalues representing about 3% of the variance, whereas distances based on dissimilarity scores give negative eigenvalues that can represent from 3 to 7% of the variance (Table 1).

**Table 1:** Comparison of scoring methods

Scoring method		% negative components
Difference	Square root	0
	Difference	3.2
Dissimilarity	BLOSUM30	4.1
	BLOSUM45	3.5
	BLOSUM62	3.6
	BLOSUM80	3.5
	PAM40	4.3
	PAM80	4.8
	PAM120	5.1
	PAM160	5.6
	PAM250	6.3
	GONNET	3.5
	JTT	6.5
	JTT_TM	6.7
	PHAT	4.1

The percent of negative components represents the weight of negative components in the variance of the data.

**Figure 2:**
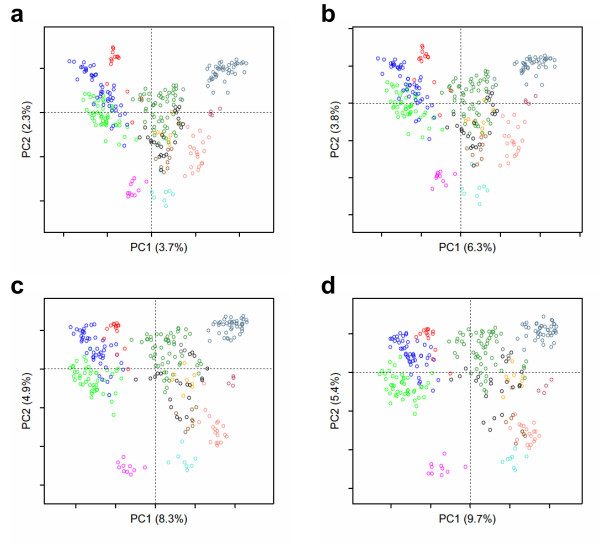
**Comparison of scoring methods.** The 2D sequence space of human GPCRs, defined by the first two components of the MDS analysis, was obtained with distances equal to the square roots of the difference scores (a), to the difference scores (b), to the dissimilarity scores calculated from the BLOSUM45 matrix (c) or from the JTT_TM matrix (d). The color code refers to the different sub-families of human GPCRs, with unclassified receptors colored in black. Plots drawn with the *mmds.2D.plot* function after coloring by GPCR sub-families with the *col.group* function.

The sequence spaces of human GPCRs obtained with the different distance matrices do not reveal dramatic differences and the overall patterns are maintained (Figure 2). In particular, the sequence spaces obtained from the difference scores or their square roots are very similar and the slight changes observed with dissimilarity scores are quite independent of the matrix used for the computation. This is illustrated in Figures 2c and d that show the 2D sequence spaces obtained with the “best” and the “worst” matrices, as defined by comparison to Euclidean distances (Table 1)

**Figure 3:**
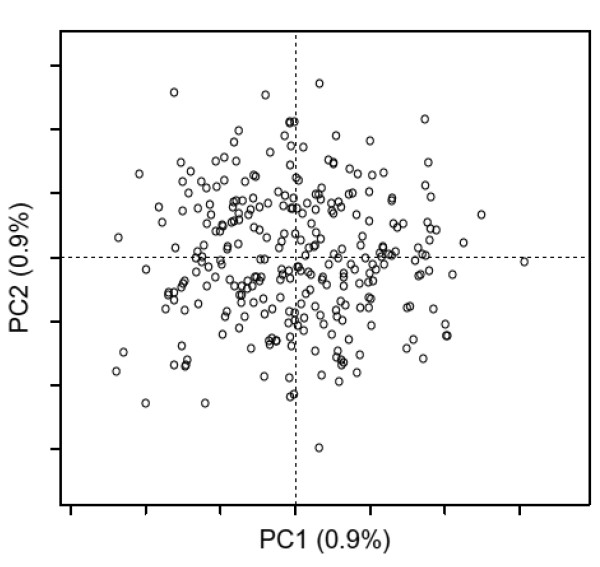
**Sequence space of random sequences.** The 2D sequence space of random sequences corresponds to the first two components of the MDS analysis of a random sequence alignment with the same properties as the human GPCR set, obtained with the *random* function. Plot drawn with the *mmds.2D.plot* function.

The “noise” of the data can be estimated from the MDS analysis of a random sequence alignment (Figure 3) that is generated with the *random* function and has the same length, number of sequences, and amino acid composition as the initial alignment.

### Comparison of orthologous sequence sets

**Figure 4:**
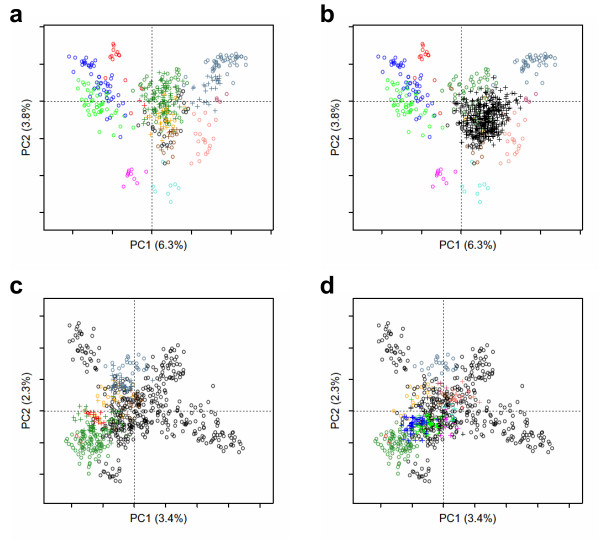
**Projection of supplementary elements.** Multiple sequence alignments of GPCRs from *H. sapiens* and *N. vectensis* were analyzed by MDS, using either human GPCRs (a, b) or anemone GPCRs (c, d) as active sequences and the other set as supplementary sequences. The active and supplementary sequences are respectively represented by dots and crosses. They are projected onto the plane defined by the first two components of the active space. The color code refers to the different sub-families of humans GPCRs, with unclassified receptors colored in black. Plots drawn with the *mmds.2D.plot* function after coloring by GPCR sub-families with the *col.group* function.

In the example shown in Figure 4, the input consists of two sets of aligned sequences [9]: the set of 283 GPCR sequences from *H. sapiens* and a set of 538 GPCR sequences from *N. vectensis* (the sea anemone). Among these anemone sequences, 139 can be assigned to five sub-families present in humans. The *mmds.project* function projects the “supplementary” sequences onto the “active” sequence space. The positions of the supplementary elements depend only on their distances to active elements. Either set can be alternatively active or supplementary. By this way, we can analyze the GPCR evolution from the point of view of humans or of sea anemones.

Figures 4a and b show the projection of the assigned and unassigned sequences from *N. vectensis*, respectively, onto the sequence space of human GPCRs. As previously discussed [9], receptors that cannot be assigned to a human sub-family are projected onto the centre of the human space since their specific evolution is expected to happen on perpendicular dimensions. The GPCR sequence space of the sea anemone has a cross shape (Figure 4c) and is driven by several large sub-families with no equivalent in humans. For clarity purpose, we project separately the human GPCRs from either “ancient” or “recent” sub-families onto this sequence space (Figures 4c and d, respectively). The “ancient” sub-families are those present both in *N. vectensis* and in *H. sapiens*[9]. The projection of the human receptors from these “ancient” sub-families onto the anemone sequence space is consistent with the reverse projection (compare Figures 4a and c). Similarly, the human receptors from “recent” sub-families, with no equivalent in *N. vectensis*, are projected towards the centre of the anemone sequence space (Figure 4d).

**Figure 5:**
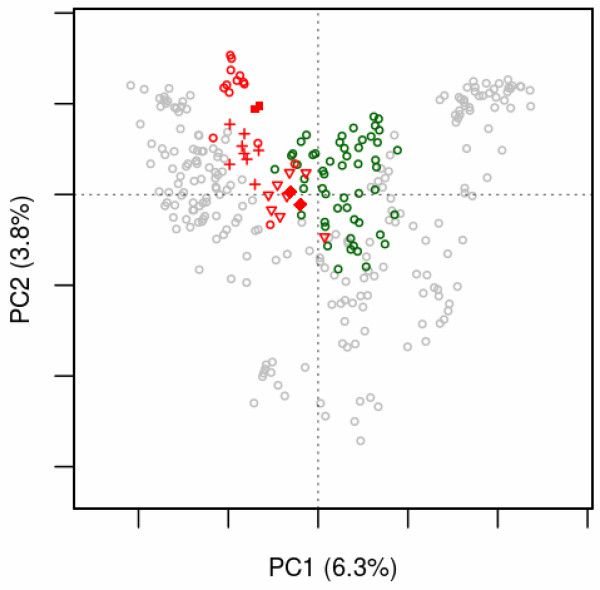
**Evolutionary drift.** The active sequence space (open circles) was obtained by the MDS analysis of the human GPCR set. Human receptors from the SO and PEP sub-families are indicated by red and green circles, respectively. The supplementary sequences correspond to SO receptors from *N. vectensis* (closed diamonds), *C. elegans* (open triangles), *C. intestinalis* (crosses) and *D. melanogaster* (closed squares). The active and supplementary sequences are projected onto the plane defined by the first two components of the active space. Plot drawn with the *mmds.2D.plot* function.

The usefulness of the projection technique is illustrated by the example of the somatostatin/opioid receptor sub-family (SO). The input consists of two sets of aligned sequences: the human set that includes 14 SO receptors, and a set of receptors from *N. vectensisC. elegansC. intestinalis* and *D. melanogaster* that could be assigned to the SO sub-family [9,33]. Figure 5 shows the projection of the orthologous SO receptors onto the sequence space of human GPCRs. We have shown previously that this sub-family initiated from a deletion in an ancestor of the peptide receptors (PEP) that led to the split between these two sub-families [33]. The SO receptors from remote species (*N. vectensis* and *C. elegans*) are close to the PEP receptors, whereas those from the chordate *C. intestinalis* and from the fruit fly *D. melanogaster* (the allatostatin receptors 1 and 2 [35]), are located at intermediary positions. Thus the projection technique reveals the evolutionary drift of specific sub-families.

## Conclusions

The R package *bios2mds* provides users with a powerful and flexible framework to perform multidimensional scaling of multiple sequence alignments. The program can import data directly as distance matrices or as multiple sequence alignments from which distance matrices are computed. The package *bios2mds* is tailored for the analysis of protein sequences but can be adapted for nucleotide sequences. Several R tools, including the *cmds* command, offer the possibility to perform MDS analysis of a distance matrix. However, to our knowledge, the *bios2mds* package is unique in allowing the projection of supplementary elements onto an active space in the R environment. This property is especially suited for the comparison of orthologous sequence sets and the evolution of specific protein sub-families. The tools for the visualization of the data have been designed to take advantage of prior knowledge on the protein family under scrutiny, for example its classification into sub-families or the presence of specific sequence motifs. Finally, it has to be emphasized that the input of the projection function within *bios2mds* requires only active and supplementary distances matrices. Thus this function can be widely used for any kind of application.

## Availability and requirements

Project name: bios2mds

Project home page: http://cran.r-project.org/web/packages/bios2mds/index.html

Operating systems: Platform independent

Programming language: R 2.12

Other requirements: requires the *amap*, *e1071*, *cluster, scales* and *rgl* packages

License: GNU General Public License

Any restrictions to use by non-academics: None

## Competing interests

The authors declare that they have no competing interests.

## Authors' contributions

JP and JMB contributed equally to this work. JP and MC conceived the package. HA provided the projection method and support to implement it in R. JP and JMB wrote the software, which JP, MC and JMB tested and debugged. MC wrote the first draft of the manuscript, which HA revised and all authors approved.
